# Pro-Oxidant Role of Silibinin in DMBA/TPA Induced Skin Cancer: ^1^H NMR Metabolomic and Biochemical Study

**DOI:** 10.1371/journal.pone.0158955

**Published:** 2016-07-14

**Authors:** Jasmine Sati, Biraja Prasad Mohanty, Mohan Lal Garg, Ashwani Koul

**Affiliations:** Department of Biophysics, Basic Medical Sciences Block, Panjab University, Chandigarh, 160014, India; UMR Inserm U1052 / CNRS 5286, FRANCE

## Abstract

Silibinin, a major bioactive flavonolignan in *Silybum marianum*, has received considerable attention in view of its anticarcinogenic activity. The present study examines its anticancer potential against 7, 12-dimethylbenz(a)anthracene (DMBA) and 12-O-tetradecanoylphorbol-13-acetate (TPA) induced skin cancer. Male LACA mice were randomly segregated into 4 groups: Control, DMBA/TPA, Silibinin and Silibinin+DMBA/TPA. Tumors in DMBA/TPA and Silibinin+DMBA/TPA groups were histologically graded as squamous cell carcinoma. In the Silibinin+DMBA/TPA group, significant reduction in tumor incidence (23%), tumor volume (64.4%), and tumor burden (84.8%) was observed when compared to the DMBA/TPA group. The underlying protective mechanism of Silibinin action was studied at pre-initiation (2 weeks), post-initiation (10 weeks) and promotion (22 weeks) stages of the skin carcinogenesis. The antioxidant nature of Silibinin was evident at the end of 2 weeks of its treatment. However, towards the end of 10 and 22 weeks, elevated lipid peroxidation (LPO) levels indicate the pro-oxidative nature of Silibinin in the cancerous tissue. TUNEL assay revealed enhanced apoptosis in the Silibinin+DMBA/TPA group with respect to the DMBA/TPA group. Therefore, it may be suggested that raised LPO could be responsible for triggering apoptosis in the Silibinin+DMBA/TPA group. ^1^H Nuclear Magnetic Resonance (NMR) spectroscopy was used to determine the metabolic profile of the skin /skin tumors. Dimethylamine (DMA), glycerophosphocholine (GPC), glucose, lactic acid, taurine and guanine were identified as the major contributors for separation between the groups from the Principal Component Analysis (PCA) of the metabolite data. Enhanced DMA levels with no alteration in GPC, glucose and lactate levels reflect altered choline metabolism with no marked Warburg effect in skin tumors. However, elevated guanine levels with potent suppression of taurine and glucose levels in the Silibinin+DMBA/TPA group are suggestive of the pro-oxidative nature of Silibinin in regressing tumors. Thus, supporting the theory of augmented LPO levels resulting in increased apoptosis in the skin tumors treated with Silibinin.

## Introduction

Nonmelanoma skin cancer (NMSC) which comprises of squamous cell carcinoma (SCC) and basal cell carcinoma (BCC) is most prevalent among Caucasians. [1] BCC accounts for 80% while SCC represents about 20% of all diagnosed NMSC cases worldwide. [2] The Indian subcontinent reports higher prevalence of SCC in comparison to BCC. [3] Apart from ultraviolet radiation (UVR), the other common causes of NMSC are environmental and occupational exposures to arsenic, polycyclic aromatic hydrocarbons (PAHs), and ionizing radiations. [4] PAHs originate during incomplete combustion of organic materials, such as wood, petroleum, coal and are known for their toxic effects besides being carcinogenic and mutagenic in nature. [5]

Current understanding of the cellular and molecular events of carcinogenesis has led to many preventive approaches such as banning of known carcinogens, targeted therapies, adopting lifestyle changes, and chemoprevention. [6] Chemopreventive agents include substances of natural or synthetic origin. Chemopreventive strategies affecting initiation, promotion, or progression play a key role in retarding or inhibiting the process of carcinogenesis. Anti-initiating strategies include scavenging DNA-reactive electrophilic compounds and free radicals, augmenting carcinogen detoxification, and stimulating DNA repair mechanisms. On the other hand, various anti-promoting and anti-progressive strategies are documented to achieve inhibition of proliferation and angiogenesis, by supporting apoptosis and scavenging of reactive oxygen species (ROS). [7] In addition to the antioxidant action of chemopreventive compounds, their pro-oxidant action in certain conditions has also been proposed to be imperative for their anti-cancer potential. [8–10] The pro-oxidant action of chemopreventive compounds through generation of ROS causes oxidative damage, membrane dysfunction, mitochondrial toxicity, and apoptotic DNA fragmentation. [11]

Potential anti-cancer properties of natural plant compounds have elicited widespread interest in recent times. [12,13] Silymarin, a polyphenol extracted from milk thistle plant (*Silybum marianum*), comprises of a concoction of at least seven active flavonolignans: Silibinin A, Silibinin B, Isosilybin A, Isosilybin B, Silychristin, Isosilychristin, Silydianin and single flavonoid Taxifolin. [14] The major and most active component of Silymarin is Silibinin (60–70%), followed by Silychristin (20%), Silydianin (10%), and Isosilybin (5%). [15] Silibinin has shown promising anti-neoplastic effects against skin, breast, lung, pancreatic, colon, cervical, prostate, bladder, and kidney carcinomas. [16]

So far, the chemopreventive effect of Silibinin has been documentated mainly on UVR induced skin cancer models. [17–19] However, the UVR induced NMSC is less common in the Asian and African population due to photoprotection by higher epidermal melanin content as compared to the Caucasian population. [20] Therefore, study on factors causing NMSC other than UVR would be more relevant for these population. In this regard, PAHs are a significant threat and several epidemiological studies emphasize a strong relationship between dermal exposure to PAHs and skin cancer. [21–24] The most commonly used PAH as an initiating agent in chemically induced skin cancer models is 7, 12 dimethylbenz(a)anthracene (DMBA). [25] DMBA and 12-O-tetradecanoylphorbol-13-acetate (TPA) induced two-stage skin cancer model has been suggested to closely mimic human SCC, which frequently metastasizes to the lymph nodes and lungs. [26] In two-stage model of skin carcinogenesis, the initiation, and promotion stages can be distinctly separated; thus, facilitating the study of stage specific response of chemopreventive compounds. [25,27] Furthermore, oncogenic Harvey rat sarcoma (H-*ras*) mutations reported in human SCCs are also caused by DMBA. [28]

In this context, the present study aims to assess a possible preventive role of Silibinin in DMBA and TPA induced two-stage skin cancer model. Various parameters like, the tumor incidence, tumor volume, tumor burden, and total number of lesions were used to evaluate the chemopreventive potential of Silibinin. Proton nuclear magnetic resonance (^1^H NMR) spectroscopy was used to determine the metabolic profile of skin/skin tumors and monitor the biochemical changes resulted from Silibinin administration. The antioxidant defense system, mode of cell death and histopathological alterations were also studied to determine the mechanism of action of Silibinin at various stages of the chemically induced skin carcinogenesis.

## Material and Methods

### Chemicals and Kits

DMBA, TPA, bovine serum albumin (BSA), 5,5-dithiobis-2-nitrobenzoic acid (DTNB), reduced glutathione (GSH), oxidized glutathione (GSSG), thiobarbituric acid (TBA), reduced nicotinamide adenine di-nucleotide phosphate (NADPH) were obtained from Sigma-Aldrich Co. (St Louis, MO, USA). Liquid chromatographic grade solvents and reagents were obtained from Merck KGaA (Darmstadt, Germany). Silibinin (M.W. 482.44) was procured from Micro Labs Limited, Bengaluru, Karnataka, India under the brand name Silybone. All other chemicals used were of analytical grade. Terminal deoxynucleotidyl transferase dUTP nick end labelling (TUNEL) assay kit was procured from Trevigen Inc. (Gaithersburg, Maryland, USA)

### Sample preparation for identification of Silibinin in plasma

Male LACA mice (25–30 g) were orally administered with a single dose of Silibinin at 500 mg/kg body weight in 0.5% carboxy methyl cellulose (CMC). Blood samples from the Silibinin treated mice were collected by retro-orbital puncture. Prior to retro-orbital puncture, the eye was anesthetized to reduce pain associated with the procedure by applying a drop of 0.5% proparacaine hydrochloride ophthalmic solution (Sunways Pvt. Ltd., India). After 5–10 seconds, excess anesthetic was blotted away with a clean gauze pad and an EDTA coated thin capillary tube was used to collect the blood into heparinized vials at 5, 10, and 30 minutes post treatment. Blood samples were centrifuged at 1500 × g for 10 minutes at 4°C to extract the plasma. The plasma was diluted at a ratio of 1:10 in methanol and further centrifuged at 1500 × g for 10 minutes at 4°C. The resulting supernatant was filtered through a 0.22 micron filter for Liquid Chromatography coupled with Mass Spectrometry (LC-MS) analysis. Additionally, plasma from untreated mice was used as control; a portion of plasma from these mice was spiked with Silibinin (1 ppm) and served as positive control. The plasma samples were analyzed in triplicate.

### Identification of Silibinin by LC-MS

Silibinin was identified in mice blood plasma using Liquid chromatography coupled with electrospray ionization mass spectrometry (LC-ESI-MS). The High Pressure Liquid Chromatography (HPLC) separation was performed at 25°C using a Waters^®^ reversed phase C18 column (100 mm × 2.1 mm dimension; 5 μm particle size). ESI-MS was carried out using Waters^®^ micromass^®^ Q-TOF Mass Spectrometer at Regional Sophisticated Instrumentation Centre (RSIC), Panjab University, Chandigarh, India. For HPLC, the mobile phase consisted of acetonitrile and 10 mM ammonium acetate (pH 5.45) in the ratio of 50:50 (v/v). The detection of Silibinin was carried out at 288 nm and the flow rate was 100 μl/min with a total run time of 10 minutes. Mass spectrometric (MS) analysis was performed in negative ion mode under the following conditions: capillary voltage 2960 V, sample cone voltage 30 V, extraction cone voltage 1.0 V and desolvation temperature 350°C. The mass spectra were recorded in the m/z range of 100–700.

### Animal model and experimental conditions

Male LACA mice weighing 25–30 g, procured from the Central Animal House, Panjab University, Chandigarh were used for the present experiment. Mice were housed in polypropylene cages bedded with sterilized rice husk and were provided standard pellet diet and water *ad libitum*. They were maintained under standard conditions of relative humidity (50–60%), temperature (24±2°C), and a 12 h dark and light cycle. Mice were allowed to acclimatize to the experimental conditions for one week and the dorsal side of mice skin was shaved using surgical clippers 2 days prior to the commencement of the various treatments. All experimental protocols were permitted by the Institutional Ethics Committee (Panjab University, Chandigarh) and were performed adhering to the doctrine of Indian National Science Academy.

**Fig 1:**
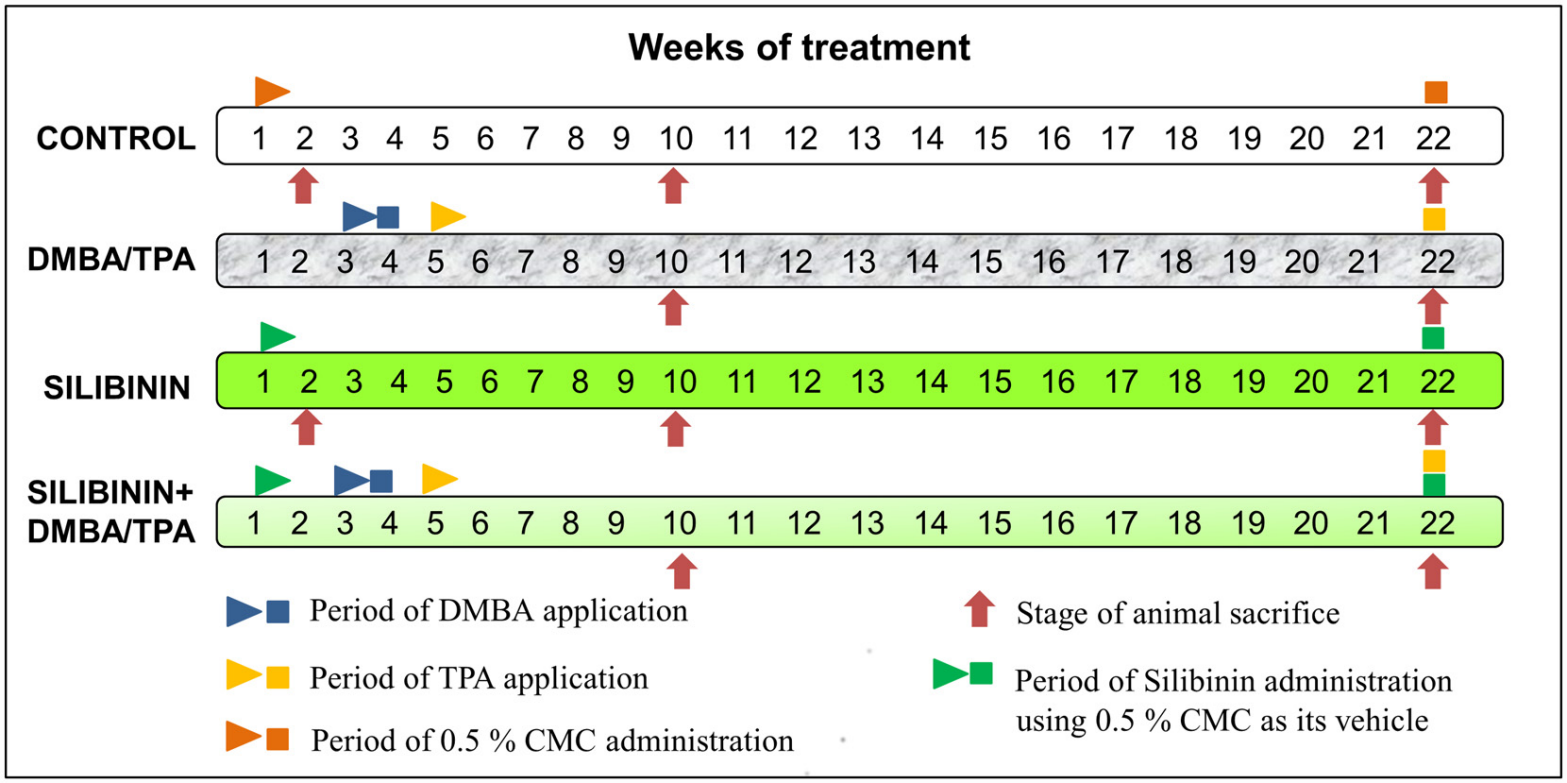
Animal treatment schedule to study the effect of Silibinin on DMBA/TPA induced skin tumorigenesis. The control group mice were gavaged with 0.5% carboxymethyl cellulose (CMC) thrice a week throughout the treatment period. In the DMBA/TPA treated group, DMBA administration was initiated from the third week of the treatment period. DMBA was topically administered at a dose of 500 nmol/100 μl of acetone twice a week for 2 weeks followed by TPA treatment (topical) at a dose of 1.7 nmol/100 μl acetone twice a week for the rest of the treatment period. In the Silibinin treated group mice, Silibinin was orally administered at a dose of 500 mg/kg body weight using 0.5% CMC as its vehicle thrice a week throughout the treatment period. In the Silibinin+DMBA/TPA group, Silibinin was administered in a similar manner as that for the Silibinin treated group. The DMBA and TPA treatment regime were identical to that of the DMBA/TPA treated group.

Mice were randomly segregated into 4 groups: Control, DMBA/TPA, Silibinin and Silibinin+DMBA/TPA comprising 10–12 animals each. Control group mice were gavaged with 0.5% CMC thrice a week throughout the treatment period. To animals of the DMBA/TPA treated group, DMBA administration was initiated from the third week of the treatment period. DMBA was topically administered at a dose of 500 nmol/100 μl of acetone twice a week for a period of 2 weeks followed by TPA treatment (topical) at a dose of 1.7 nmol/100 μl acetone twice a week through the rest of the treatment period. The Silibinin group mice were orally administered with Silibinin at a dose of 500 mg/kg body weight using 0.5% CMC as its vehicle thrice a week throughout the treatment period. [29] In the Silibinin+DMBA/TPA group mice, Silibinin was administered in a similar manner as that for the Silibinin treated group. The DMBA and TPA treatment regime was identical to that of the DMBA/TPA treated group. The animal treatment schedule is illustrated in Fig 1. The body weight, diet and water consumption were observed every week in all the groups throughout the treatment period. Additionally, the tumor incidence and the number of papilloma’s were also recorded weekly. The mice were sacrificed by cervical dislocation during the process of skin tumorigenesis.

### Tumor assessment

At the end of the treatment period (22 weeks), the skin/skin tumors were excised and analyzed for (a) percentage of tumor incidence (number of animals showing carcinogenic response / total number of animals in the group × 100) (b) mean tumor volume (4/3 πr^3^ where r is mean tumor radius in mm) (c) mean tumor burden (mean tumor volume × mean number of tumors) (d) total number of lesions. [30]

### Biochemical Studies

In order to investigate the response of Silibinin administration on the extent of oxidative stress and the status of antioxidant enzymes at pre-initiation, post-initiation and promotion stages of skin carcinogenesis, the animals were sacrificed at the end of 2, 10 and 22 weeks respectively. Tris base (0.2 M, pH 7.4) was used to make 20% homogenate (w/v) of skin/skin tumors by means of electrically driven Teflon fitted Potter Elveihem homogenizer. A small portion of the tissue homogenate was kept at 4°C to determine the GSH levels. The post mitochondrial fraction (PMF) was prepared by centrifugation of the tissue homogenates at 10,000 × g for 30 minutes at 4°C for the estimation of activity of superoxide dismutase (SOD), catalase (CAT), glutathione reductase (GR), glutathione peroxidase (GPx), and the levels of lipid peroxidation (LPO).

### Assessment of Oxidative Stress

#### Extent of peroxidative damage

The quantitative measurement of LPO was performed using the method of Trush *et al*. [31] Malondialdehyde (MDA), a degradation product of peroxidized lipids, is considered as an index of LPO. MDA reacts with TBA to generate the MDA-TBA chromophore, which can be quantified colorimetrically at 532 nm. The results were expressed as nanomoles of MDA-TBA chromophore formed per milligram of protein, using the molar extinction coefficient of 1.56 x 10^5^ M^-1^ cm^-1^.

#### Assessment of GSH levels

GSH is estimated as the total non-protein sulfhydryl group that reacts with DTNB and forms a yellow colored complex that absorbs at 412 nm as described by Moron *et al*. [32] GSH was used as a standard to calculate nanomoles of GSH per milligram of protein.

#### Estimation of SOD activity

The activity of SOD was estimated by the method described by Kono. [33] It is based on the inhibitory effect of SOD on the reduction of nitro blue tetrazolium (NBT) to blue coloured formazan by superoxide anions (measured at 560 nm), which were generated by the photo-oxidation of hydroxylamine hydrochloride. The SOD activity was expressed as International Units per mg protein (IU/mg protein), which is inverse of the amount of protein required to inhibit the reduction rate of NBT by 50%.

#### Estimation of CAT activity

The CAT activity was determined by measuring the decomposition of H_2_O_2_ into water and oxygen at 240 nm as reported by Luck. [34] One unit of enzyme activity was expressed as IU/mg protein.

#### Estimation of GPx activity

The GPx activity was measured by the method reported by Flohe and Gunzler. [35] GSH is oxidized to GSSG upon reduction of H_2_O_2_ by GPx. The GSSG produced is then recycled back to GSH by GR using NADPH. The oxidation of NADPH to NADP^+^ is accompanied by decrease in absorbance at 340 nm. The rate of decrease in A_340_ is directly proportional to the GPx activity in the sample. One unit of enzyme activity was expressed as nmol of NADPH consumed/min/mg protein using the extinction coefficient of 6.22 mM^-1^cm^-1^.

#### Estimation of GR activity

The GR activity was based on the NADPH dependent reduction of GSSG to GSH as described by Williams and Arscott. [36] The oxidation of NADPH to NADP^+^ was accompanied by the decrease in A _340_ and was directly proportional to the activity of GR in the sample. One unit of enzyme activity was expressed as nmol of NADPH consumed/min/mg protein using the extinction coefficient of 6.22 mM^-1^cm^-1^.

#### Estimation of protein content

The protein content of homogenates and PMF was determined using BSA as a standard at 620 nm by the method of Lowry *et al*. [37]

### Histopathology

The skin/skin tumors were excised at the end of 10 and 22 weeks, fixed in 10% formaldehyde in phosphate buffer saline (pH 7.4) for hematoxylin and eosin (H&E) staining. The tissues were briefly dehydrated in increasing grades of alcohol, cleared in benzene and embedded in low melting point paraffin wax. 5 μm thick sections were cleaved and placed on a clean glass slide. The tissue sections were deparaffinized in decreasing grades of alcohol. For each tissue, at least three adjoining sections were prepared and stained with H&E for assessment under light microscope (LEICA DM 3000).

### TUNEL Assay

The TUNEL assay was performed following the culmination of the treatment period (22 weeks) on deparaffinised and rehydrated tissue sections for the detection of DNA fragmentation using TACS-XL DAB in situ apoptosis detection kit. The DNA fragmentation in the apoptotic cells was identified by addition of brominated nucleotides (BrdU) using terminal deoxynucleotidyl transferase (TdT) onto the free 3′OH residue of the DNA fragments. The incorporated BrdU was detected using a highly specific biotinylated anti-BrdU antibody. Biotin labeled fragments were identified by incubation with streptavidin horseradish peroxidase and subsequently with 3, 3′-diamino benzidine. The slides were counter stained with methyl green for better background details and observed under light microscope. The number of TUNEL (+ve) cells were quantified over the total number of cells in the selected tissue section. The apoptotic index was expressed in percentage.

### Sample preparation for ^1^H NMR spectroscopy

Precisely weighed skin/skin tumors (300 mg), excised immediately after animal sacrifice, were flash frozen in liquid N_2,_ and stored at -40°C. The frozen tissue samples were finely ground in liquid nitrogen using pre-cooled pestle and mortar. Solvent extraction of metabolites from the ground tissue samples was performed using the methanol-chloroform-water extraction procedure by Beckonert *et al*. [28] with some modifications. Briefly, the ground tissue sample was mixed with 4 ml of chloroform: methanol (1:1 v/v) and vortexed for 1 minute. The mixture was then kept at -20°C for 30 minutes to precipitate the proteins. Then, 2 ml of ice-cold distilled water was added to it and centrifuged at 10,000 × g for 15 minutes. The lower organic phase (chloroform) and upper aqueous phase (methanol/water) were clearly separated by an insoluble interface. The aqueous and organic phases were transferred by pipetting into separate vials. The insoluble interface was extracted again with the same set of solvents and the second set of fractions obtained were pooled with the first one to enhance recovery. The aqueous phase was freeze-dried and reconstituted in 750 μl of deuterium oxide (D_2_O) (pH 7.4) containing trimethylsilylpropionic acid (TMSP-d_4_) at a concentration of 0.5 mM and transferred into 5 mm NMR tubes.

### Quantitative ^1^H-NMR spectroscopy of skin tissue extracts

One dimensional NMR spectra were obtained on a Bruker 400 MHz DRX spectrometer equipped with Bruker Topspin 1.3 software (Bruker BioSpin, Rheinstetten, Germany) using an inverse 5 mm TXI probe. For metabolites quantification, a standard water presaturation pulse program “zgpr” was used to suppress water residue signal. An internal standard, TMSP-d_4_ was used for chemical shift referencing at 0 ppm. The intensity of the TMSP peak was used to determine the concentration of each metabolite. The spectral analysis and metabolite signal integration was carried out using the ACD NMR processor software (Advanced Chemistry Development Inc., Toronto, Canada). The ^1^H-NMR peaks for each metabolite was identified using Human Metabolome Database (HMDB) [38] and data obtained from various research papers. [27,39–41]

### Statistical Analysis

The data were represented as mean ± S.D and examined with Student’s t-test for the tumor statistics and one-way analysis of variance (ANOVA) using the SPSS software package (version 16) for Windows (SPSS Inc., Chicago, IL) to analyze the biochemical parameters. Principal Component Analysis (PCA) was applied to the hydrophilic metabolites identified from the ^1^H NMR spectrum using web-based metabolomic data processing tool MetaboAnalyst 3.0 [42]

## Results

### Determination of Silibinin in Plasma

The absorption of orally administered Silibinin (500 mg/kg body weight) in the systemic circulation was determined by performing LC-ESI-MS. In the negative ion mode, the MS profile of Silibinin (M.W. 482.44) gives the most dominant peak at m/z 481 corresponding to the [M-H]^-1^ ion; where M stands for Silibinin. The liquid chromatographic (LC) profile of 50 ppm Silibinin solution is shown in S1(A) Fig In this figure, the peaks from 2 diastereoisomers of Silibinin are well resolved with retention time of 3.18 and 4.12 minutes. Due to the time lag between the ultra violet (UV) absorption measurement during LC and the ionization of MS, the total ion current (TIC) profile of MS at 3.46 and 4.46 minutes (S1(B) Fig) correspond to the peaks at 3.18 and 4.12 minutes in the LC chromatogram respectively. The electrospray ionisation mass spectrometry (ESI-MS) spectra of 50 ppm Silibinin solution shows intense peak at m/z 481 corresponding to the LC retention time of 3.18 minutes (S1(C) Fig) and 4.12 minutes (S1(D) Fig) respectively, thus validating the elution and separation of Silibinin isomers. The MS spectra presented in the S1–S6 Figs are generated by summing up the MS scans of 0.5 minutes (0.25 minutes on each side of the centroid of LC peak). S2–S6 Figs depict typical LC-ESI-MS spectra of the plasma from untreated mice (control), plasma from mice blood spiked with 1 ppm Silibinin (positive control), blood samples collected after single Silibinin dose (500 mg/kg body weight, per oral) at 5 minutes, 10 minutes and 30 minutes post treatment respectively. No significant peaks were observed in the m/z region 460 to 500 of the MS spectra of plasma from untreated animals (S2 Fig). The MS spectra of plasma from mice blood sample spiked with 1 ppm Silibinin (S3 Fig) display a peak at m/z 480 along with the peak at m/z 481. The peak at m/z 480 originates due to the dissociation of Silibinin conjugates, which are formed in the blood plasma, during electrospray ionization process. In the blood samples of mice at 5 and 30 minutes post treatment, no signature peaks of Silibinin were observed (m/z 480 and 481). However, in the blood samples collected from mice 10 minutes post treatment, signature peak of Silibinin conjugate (m/z 480) was observed thus, confirming the absorption of drug in the blood plasma. The identification of Silibinin in the plasma of mice at 10 minutes post treatment is in accordance with the previous reports. [15,43]

### Dietary intake and body weights

**Fig 2:**
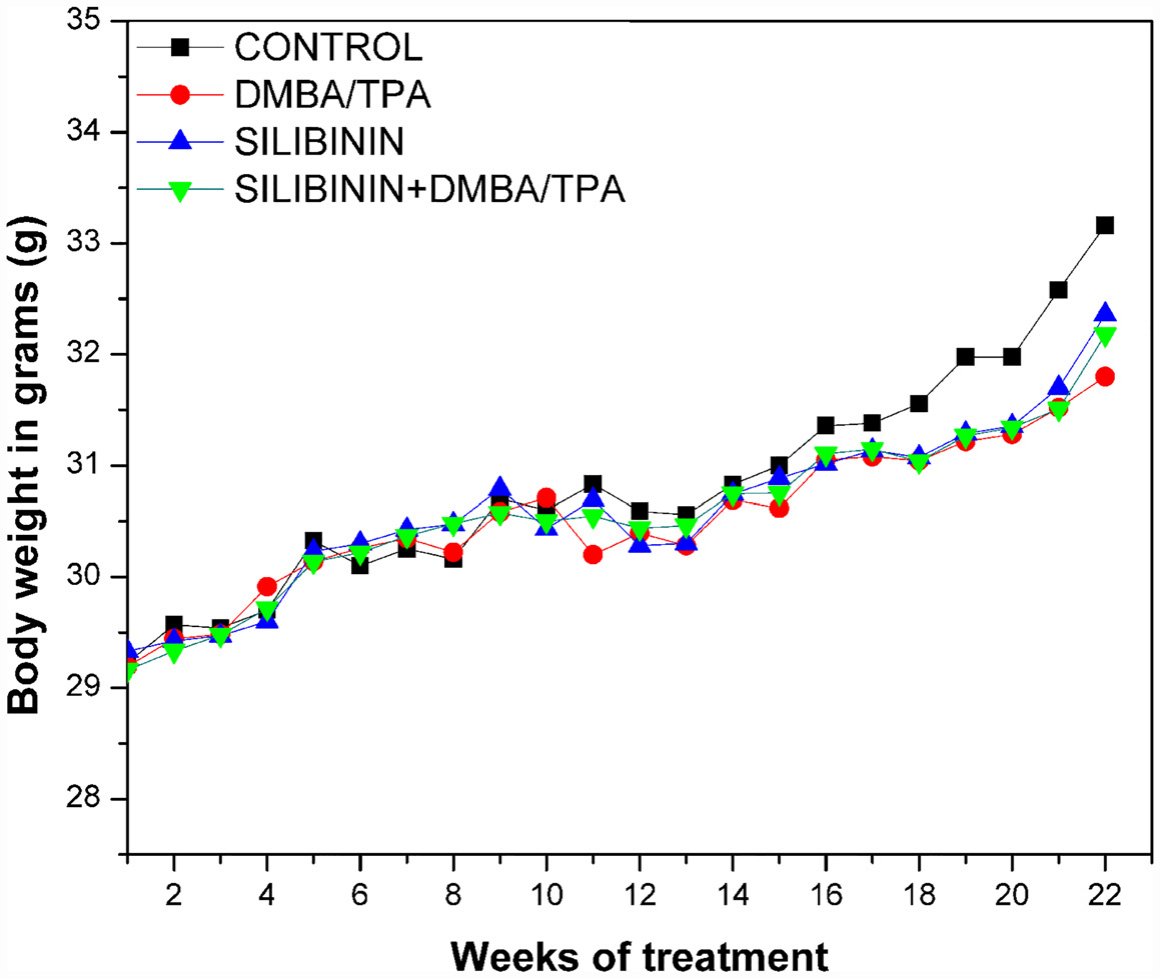
The average body weight of the animals in each group (10–12 mice/group) measured after the end of each week till the end of the treatment period.

The mice in all the treated groups displayed no substantial change in the body weight, diet, and water consumption during the experimental time period. The average body weight of the animals was recorded each week and has been plotted as a function of time in Fig 2.

### Tumor statistics

**Fig 3:**
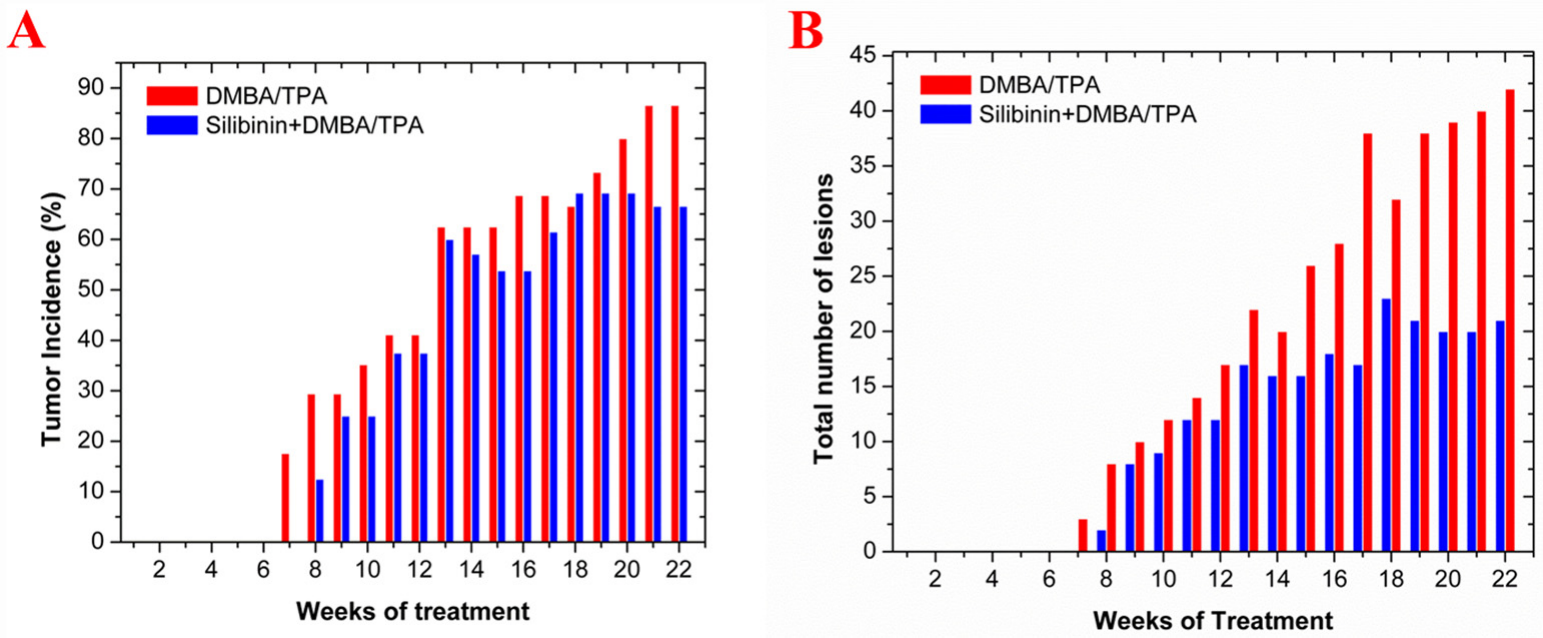
Effect of Silibinin on the DMBA/TPA induced skin tumorigenesis in LACA mice. In the DMBA/TPA treated group, DMBA administration was commenced from the third week of the treatment period. DMBA was topically administered at a dose of 500 nmol/100 μl of acetone twice a week for 2 weeks followed by TPA treatment (topical) at a dose of 1.7 nmol/100 μl acetone twice a week for the rest of the treatment period. In the Silibinin+DMBA/TPA group mice, Silibinin was orally administered at a dose of 500 mg/kg body weight using 0.5% CMC as its vehicle thrice a week throughout the treatment period. The DMBA and TPA treatment regime were identical to that of the DMBA/TPA treated group. **(A**) The tumor incidence i.e. the percentage of mice with tumors and **(B)** total number of lesions in the DMBA/TPA and Silibinin+DMBA/TPA groups mice were plotted with respect to the number of weeks of treatment. The tumor incidence and total number of lesions are from 10–12 mice/group.

At first, the chemopreventive efficacy of Silibinin against DMBA/TPA induced skin tumorigenesis was evaluated at the end of the treatment period using tumor incidence, tumor volume and tumor burden parameters. Papillomas first appeared in the DMBA/TPA group during the sixth week of treatment whereas, in case of the Silibinin+DMBA/TPA group, their first appearance was delayed by one week (i.e. seventh week). The tumor incidence was 86.6% in the DMBA/TPA and 66.6% in the Silibinin+DMBA/TPA group with a protective effect of about 23% (Fig 3). Also, there was a marked decrease (p<0.01) in the mean tumor volume of the Silibinin+DMBA/TPA group (55.1±21.4 mm^3^) as compared to the DMBA/TPA group (155±121 mm^3^) (Table 1). In DMBA/TPA group, maximum volume observed was 409 mm^3^ whereas in Silibinin+DMBA/TPA group, the maximum volume observed was 72.6 mm^3^. Similarly, the mean tumor burden was significantly lowered (p<0.01) in the Silibinin+DMBA/TPA (102±39.7 mm^3^) as compared to the DMBA/TPA group (672±524 mm^3^) (Table 1). The total number of lesions were found to be 42 in the DMBA/TPA group and 21 in the Silibinin+DMBA/ TPA group (Fig 3).

**Table 1:** Modulatory role of Silibinin on DMBA/TPA induced skin tumors.

Parameters	Test Groups			
	Control	DMBA/TPA	Silibinin	Silibinin+DMBA/TPA
**Tumor incidence**	Nil	86.6%	0%	66.6%
**Mean tumor volume (mm**^**3**^**)**	Nil	155±121; Range: 33.4–409	Nil	55.1±21.4^##^; Range: 23.9–72.6
**Mean tumor burden (mm**^**3**^**)**	Nil	672±524; Range: 144–1770	Nil	102±39.7^##^; Range: 44.2–134

Data are analyzed using Student’s t-test and expressed as mean±SD (n = 10–12 animals/group).

^##^ (p<0.01) indicate significant changes with respect to the DMBA/TPA treated group.

### Biochemical Parameters

Since, Silibinin is known to possess antioxidant properties, the implication of its antioxidative role in prevention of skin carcinogenesis have been ascertained through the analysis of various biochemical parameters. Lipid peroxidation along with various enzymatic and non-enzymatic antioxidants have been quantified for this assessment. The peroxidation of lipids is the most detrimental process observed in every living organism. Oxidative stress is determined by measuring the levels of LPO, an established marker of oxidative damage. GSH, a non-enzymatic antioxidant is the most abundant thiol compound found in mammalian cells. The antioxidant enzymes such as GPx, GR, SOD and CAT form the body’s endogenous defense mechanism to protect against free radical induced cell damage. GPx catalyzes the reduction of peroxides by using GSH as the electron donar whereas GR recycles the oxidized glutathione (GSSG) to GSH by using NADPH as the electron donar. SOD enzyme is involved in the conversion of superoxide ions into hydrogen peroxide and oxygen while CAT catalyzes the breakdown of hydrogen peroxide into water and oxygen. These biochemicals were estimated at various stages of skin carcinogenesis, namely the pre-initiation (2 weeks), the post-initiation (10 weeks) and the promotion (22 weeks) stages, and the results are described below.

#### Pre-initiation stage of skin carcinogenesis

After 2 weeks of treatment period, the antioxidant defense system was assessed by evaluating several biochemical parameters listed in Table 2. The LPO levels were significantly lower (p<0.001) in the Silibinin treated group than in the control group. There was no appreciable difference in the GSH levels and GR activity in between the Silibinin group and the control group. In comparison to the control group, the GPx, CAT and SOD activities were found to be upregulated significantly (p<0.01) in the Silibinin group.

**Table 2:** Effect of Silibinin on the extent of oxidative stress and antioxidant defense system in skin of LACA mice at the pre-initiation stage of skin carcinogenesis.

Parameters	Test Groups	
	Control	Silibinin
**Lipid peroxidation**^1^ **(LPO)**	3.15±0.52	0.92±0.18***
**Reduced glutathione**^2^ **(GSH)**	43.6±5.37	41.7±5.50
**Glutathione peroxidase**^3^ **(GPx)**	13.8±4.08	20.6±2.24**
**Glutathione reductase**^4^ **(GR)**	4.64±1.15	4.41±1.35
**Catalase**^5^ **(CAT)**	27.1±5.44	54.2±9.18**
**Superoxide dismutase**^6^ **(SOD)**	11.1±1.38	16.4±1.32**

^1^nmol of MDA-TBA chromophore formed/mg protein,

^2^nmol GSH/mg protein,

^3^nmol NADPH oxidized/min/mg protein,

^4^nmol NADPH oxidized/min/mg protein,

^5^IU/mg protein,

^6^IU/mg protein.

Data are expressed as mean±SD and are analyzed by one-way ANOVA followed by post hoc test.

** (p<0.01),

*** (p<0.001) indicate significant change with respect to the control group.

#### Post-initiation stage of skin carcinogenesis

After 10 weeks of treatment period, the GSH, LPO levels and activities of GPx, GR, CAT and SOD were estimated and tabulated in Table 3. The GSH, LPO levels as well as the activities of GPx, GR, CAT, and SOD were unaltered in the DMBA/TPA group in comparison to the control group. There was no difference in the GSH, LPO levels as well as the activities of GPx, GR and CAT in between the Silibinin and control groups. However, the SOD activity was significantly higher (p<0.01) in Silibinin group than that in the control group. In the Silibinin+DMBA/TPA group, there was a significant decline in the activities of GPx (p<0.01) and SOD (p<0.05) than the DMBA/TPA group.

**Table 3:** Effect of Silibinin on the extent of oxidative stress and antioxidant defense system in skin of LACA mice at the post-initiation stage of skin carcinogenesis.

Parameters	Test Groups			
	Control	DMBA/TPA	Silibinin	Silibinin+DMBA/TPA
**Lipid peroxidation**^1^ **(LPO)**	3.09±0.71	3.39±0.38	2.63±0.33	4.31±0.39**^††^
**Reduced glutathione**^2^ **(GSH)**	44.7±7.90	30.5±2.80	38.6±0.90	26.5±2.10***^†††^
**Glutathione peroxidase**^3^ **(GPx)**	14.3±2.28	14.2±2.10	12.9±1.70	10.8±0.69**^##^
**Glutathione reductase**^4^ **(GR)**	4.32±1.32	4.71±1.32	3.93±0.39	2.85±0.18^#^
**Catalase**^5^ **(CAT)**	26.5±1.70	33.1±1.10	30.5±4.90	38.8±8.70
**Superoxide dismutase**^6^ **(SOD)**	10.2±2.20	12.9±0.50	16.5±1.80**	10.6±1.35^††^

^1^nmol of MDA-TBA chromophore formed/mg protein,

^2^nmol GSH/mg protein,

^3^nmol NADPH oxidized/min/mg protein,

^4^nmol NADPH oxidized/min/mg protein,

^5^IU/mg protein,

^6^IU/mg protein. Data are expressed as mean±SD and are analyzed by one-way ANOVA followed by post hoc test.

** (p<0.01),

*** (p<0.001) indicate significant change with respect to the control group;

^#^ (p<0.05),

^##^ (p<0.01) indicate significant change with respect to the DMBA/TPA treated group;

^††^ (p<0.01),

^†††^ (p<0.001) indicate significant change with respect to the Silibinin treated group.

#### Promotion stage of skin carcinogenesis

In Table 4, the GSH, LPO levels, and activities of GPx, GR, CAT, and SOD estimated after 22 weeks of treatment are summarized. In the DMBA/TPA group, a significant decrease in the LPO levels (p<0.001) as well as attenuation in the activity of antioxidant enzymes, GPx (p<0.01), GR (p<0.001) and SOD (p<0.001) was observed in comparison to the control group. There was no significant change in the GSH levels and the CAT activity in the DMBA/TPA group with respect to the control group. The GSH, LPO levels and the activity of antioxidant enzymes (GPx, GR, CAT, SOD) remain statistically unaltered in the Silibinin treated group as compared to the control group. In the Silibinin+DMBA/TPA group, significant enhancement in the LPO and GSH levels as well as upregulation of GR activity was observed in comparison to the DMBA/TPA group. There was no appreciable change in the GPx, CAT and SOD activity in the Silibinin+DMBA/TPA group in comparison to the DMBA/TPA group.

**Table 4:** Effect of Silibinin on the extent of oxidative stress and antioxidant defense system in skin of LACA mice at the promotion stage of skin carcinogenesis.

Parameters	Test Groups			
	Control	DMBA/TPA	Silibinin	Silibinin+DMBA/TPA
**Lipid peroxidation**^1^ **(LPO)**	3.74±0.75	1.49±0.32***	4.02±0.83^###^	3.83±0.71^###^
**Reduced glutathione**^2^ **(GSH)**	32.7±6.27	28.1±3.75	27.1±5.23	35.5±4.56^###^^†††^
**Glutathione peroxidase**^3^ **(GPx)**	11.7±2.20	7.78±1.46**	10.7±2.26^###^	8.44±0.52***
**Glutathione reductase**^4^ **(GR)**	5.67±0.75	3.60±0.39***	5.21±0.36^###^	4.35±0.41**^###^^†††^
**Catalase**^5^ **(CAT)**	39.1±3.31	38.2±7.21	35.1±6.05	37.6±2.17
**Superoxide dismutase**^6^ **(SOD)**	12.8±2.07	5.96±0.64***	12.1±2.42^###^	6.94±1.35***^†††^

^1^nmol of MDA-TBA chromophore formed/mg protein,

^2^nmol GSH/mg protein,

^3^nmol NADPH oxidized /min/mg protein,

^4^nmol NADPH oxidized /min/mg protein,

^5^IU/mg protein,

^6^IU/mg protein. Data are expressed as mean±SD and are analyzed by one-way ANOVA followed by post hoc test.

** (p<0.01),

*** (p<0.001) indicate significant change with respect to the control group;

^###^ (p<0.001) indicate significant change with respect to the DMBA/TPA treated group;

^†††^ (p<0.001) indicate significant change with respect to the Silibinin treated group.

### Histopathology

**Fig 4:**
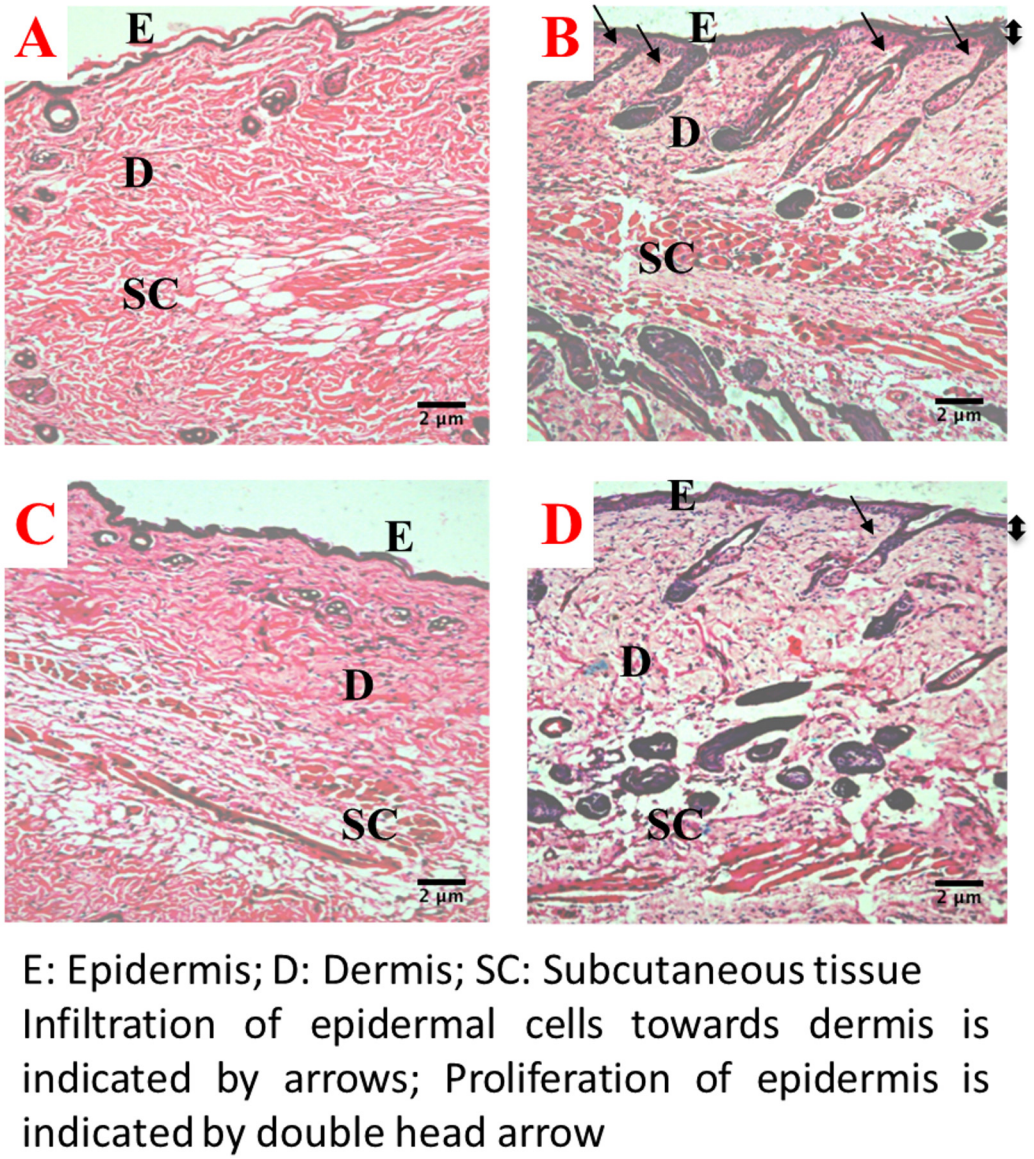
Histopathology of skin/skin tumors in various treatment groups towards the end of 10 weeks at 100 X magnification. (A) and (C) represent normal skin histoarchitecture from the control and Silibinin treated groups respectively:exhibiting well defined epidermis (E), underlying dermis (D) and subcutaneous tissue (SC); (B) and (D) represent DMBA/TPA and Silibinin+DMBA/TPA treated groups respectively: the proliferation of epidermis is indicated by the double headed arrow while the infiltration of epidermal cells towards dermis is marked with single headed arrow.

**Fig 5:**
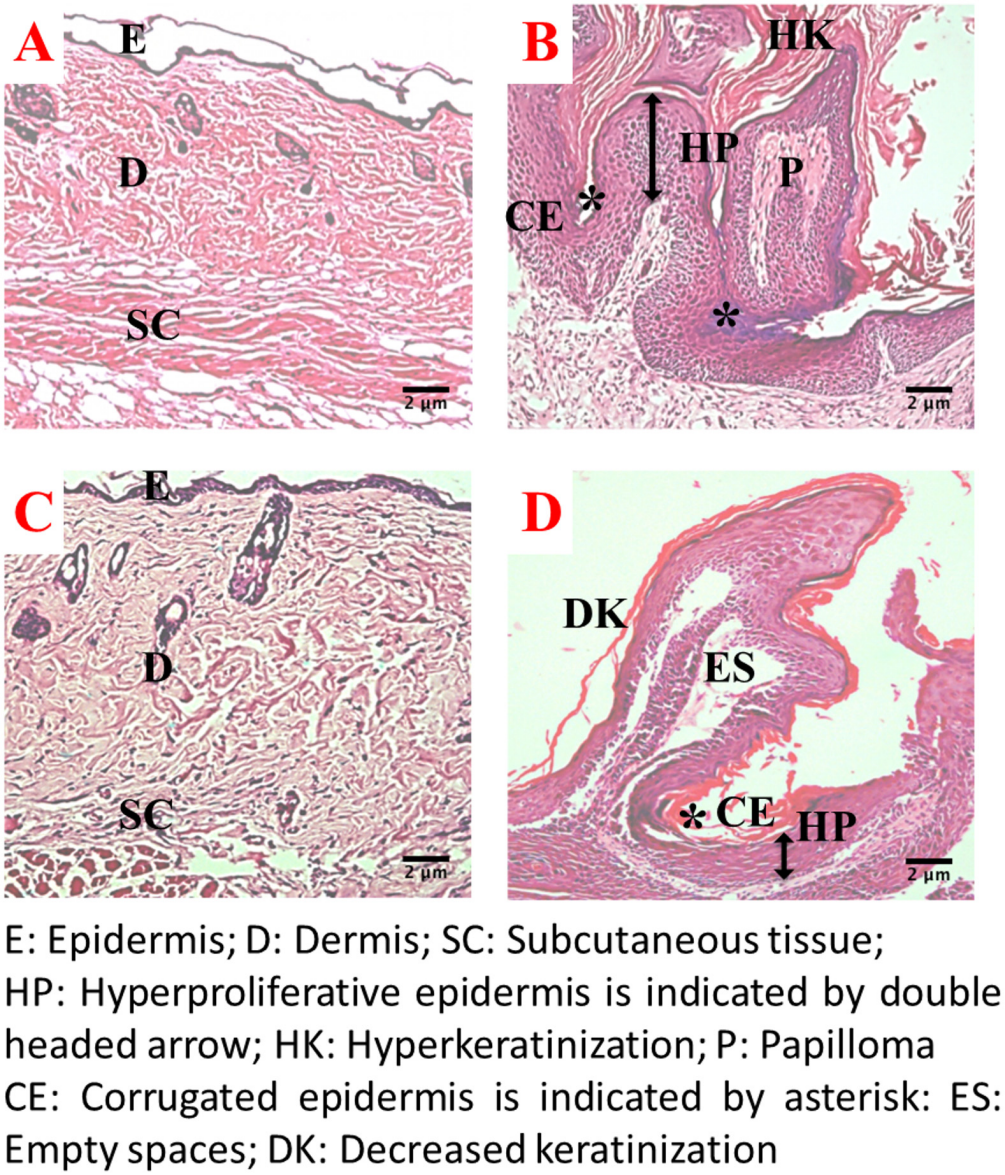
Histopathology of skin/skin tumors in various treatment groups towards the end of 22 weeks at 100 X magnification. (A) and (C) represent normal skin histoarchitecture from the control and Silibinin treated groups respectively:exhibiting well defined epidermis (E), underlying dermis (D) and subcutaneous tissue (SC); (B) aberrant histoarchitecture displaying corrugated epidermis (CE) indicated by asterisk, hyperproliferative epidermis (HP) indicated by double headed arrow, hyperkeratinization (HK) and well developed papilloma (P) from the DMBA/TPA treated group; (D) hyperproliferative epidermis (HP) with decreased keratininization (DK) and empty spaces (ES) in lumen of tumor were prominent features observed from Silibinin+DMBA/TPA treated group.

The histopathological analysis of the skin/skin tumors were carried out at the end of 10 and 22 weeks (Figs 4 and 5). The skin of the control group mice exhibited well-defined epidermis, underlying dermis, and subcutaneous tissue. At the end of 10 and 22 weeks, the skin of Silibinin treated mice were similar to that of the control mice with mild inflammation of the epidermis. DMBA/TPA treatment to the depilated skin of mice at the end of 10 weeks induced epidermal proliferation with the invasion of the epidermal cells towards the dermis. The intervention of Silibinin to DMBA/TPA treated skin also resulted in epidermal thickening but the extent of infiltration of the epidermal cells was much less (Fig 4). At the end of 22 weeks (Fig 5), the DMBA/TPA treatment resulted in well-developed SCC with hyperchromatia, thickened corrugated epidermis and hyperkeratosis as prominent features. In the Silibinin+DMBA/TPA treated skin tumors, empty spaces with considerably less keratinization around the epidermis was observed. In addition, the amount of hyperchromatia was remarkably lower in the Silibinin+DMBA/TPA treated group in comparison to the DMBA/TPA group.

### TUNEL assay

**Fig 6:**
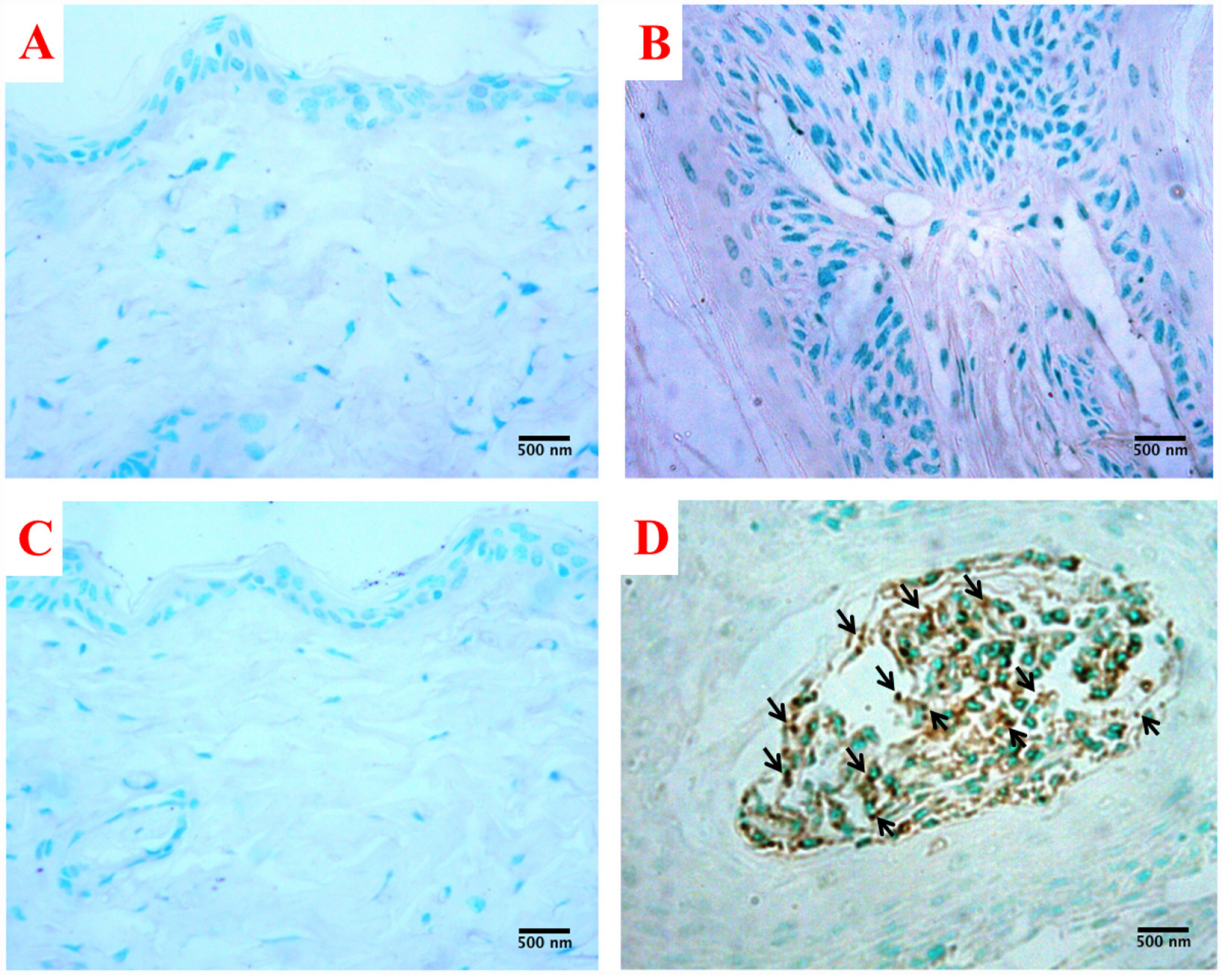
Effect of DMBA/TPA and/or Silibinin on apoptotic index through TUNEL assay in skin/skin tumors at 400 X magnification. (A) and (C) represent Control and Silibinin treated groups displaying the normal histoarchitecture as blue-green stained cells; (B) DMBA/TPA treated mouse exhibiting cellular proliferation as depicted by the large number of darkly stained blue green cells; (D) Silibinin+DMBA/TPA treated mouse exhibits a cluster of brown stained apoptotic cells in the lumen of the tumor (arrowed). Data are expressed as mean±SD (n = 5) and are analyzed by one-way ANOVA followed by post hoc test. ***p<0.001 indicate significant change with respect to the control group; ^###^p<0.001 indicate significant change with respect to the DMBA/TPA group; ^†††^p<0.001 indicate significant change with respect to the Silibinin treated group.

In order to determine the effect of Silibinin on apoptosis in skin/skin tumors, TUNEL assay was performed at the end of the treatment period. The quantitative analysis of TUNEL positive cells (brown stained cells) against normal cells (blue green stained cells) in random microscopic fields of tissue section were carried out in different experimental groups. The number of apoptotic cells were significantly higher (p<0.001) in the Silibinin+DMBA/TPA treated group in comparison to the control, DMBA/TPA and Silibinin treated groups (Fig 6; Table 5).

**Table 5:** Effects of Silibinin on the apoptotic index through TUNEL assay in skin/skin tumors at the promotion stage of skin carcinogenesis.

Parameter	Test Groups			
	Control	DMBA/TPA	Silibinin	Silibinin+DMBA/TPA
**Apoptotic index (%)**	2.80±0.33	1.75±0.04	2.78±0.46	18.0±1.58***^###^^†††^

Data are expressed as mean±SD and are analyzed by one-way ANOVA followed by post hoc test.

*** (p<0.001) indicate significant change with respect to the control group;

^###^ (p<0.001) indicate significant change with respect to the DMBA/TPA treated group;

^†††^ (p<0.001) indicate significant change with respect to the Silibinin treated group.

### Spectral profile and Statistical analysis of NMR data

**Fig 7:**
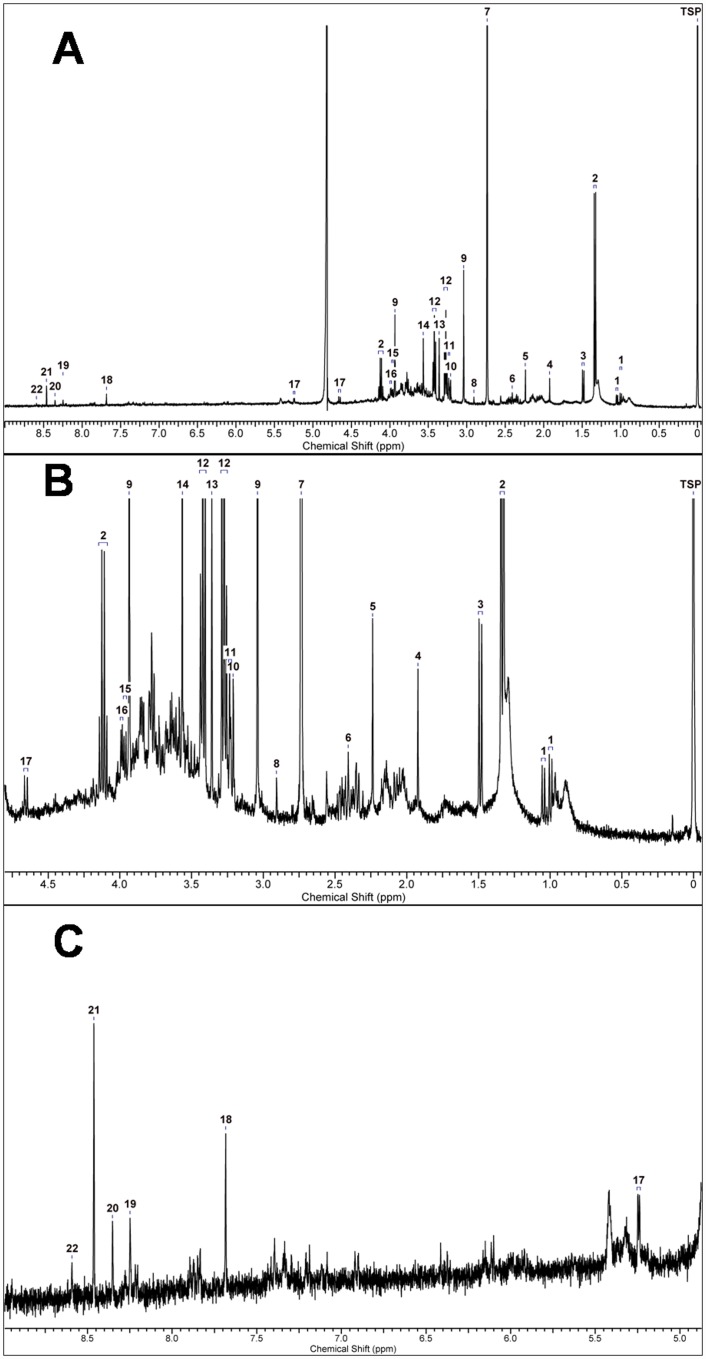
^1^H nuclear magnetic resonance spectrum of hydrophilic extract of skin tissue from mouse of control group. (A) The ^1^H NMR spectrum in the chemical shift region of 0 to 10 ppm. Expanded views of the above spectrum in the chemical shift range of; (B) 0 to 4.75 ppm and (C) 5.0 to 9.0 ppm are also shown. The metabolites assigned to different peaks are 1, Valine; 2, Lactate; 3, Alanine; 4, Acetate; 5, Acetone; 6, Succinate; 7, Dimethylamine (DMA); 8, Unknown 1; 9, Creatine; 10, Choline; 11, Unknown 2; 12, Taurine; 13, Glycerophosphocholine (GPC); 14, Glycine; 15, Unknown 3; 16, Unknown 4; 17, Glucose;18, Guanine; 19, Unknown 5; 20, Unknown 6; 21, Formate; 22, Unknown 7.

In the ^1^H NMR analysis, 26 distinct peaks were identified out of which 19 peaks corresponded to 15 known metabolites. There was ambiguity in the identification of seven peaks and were referred as unknown (Fig 7).

**Fig 8:**
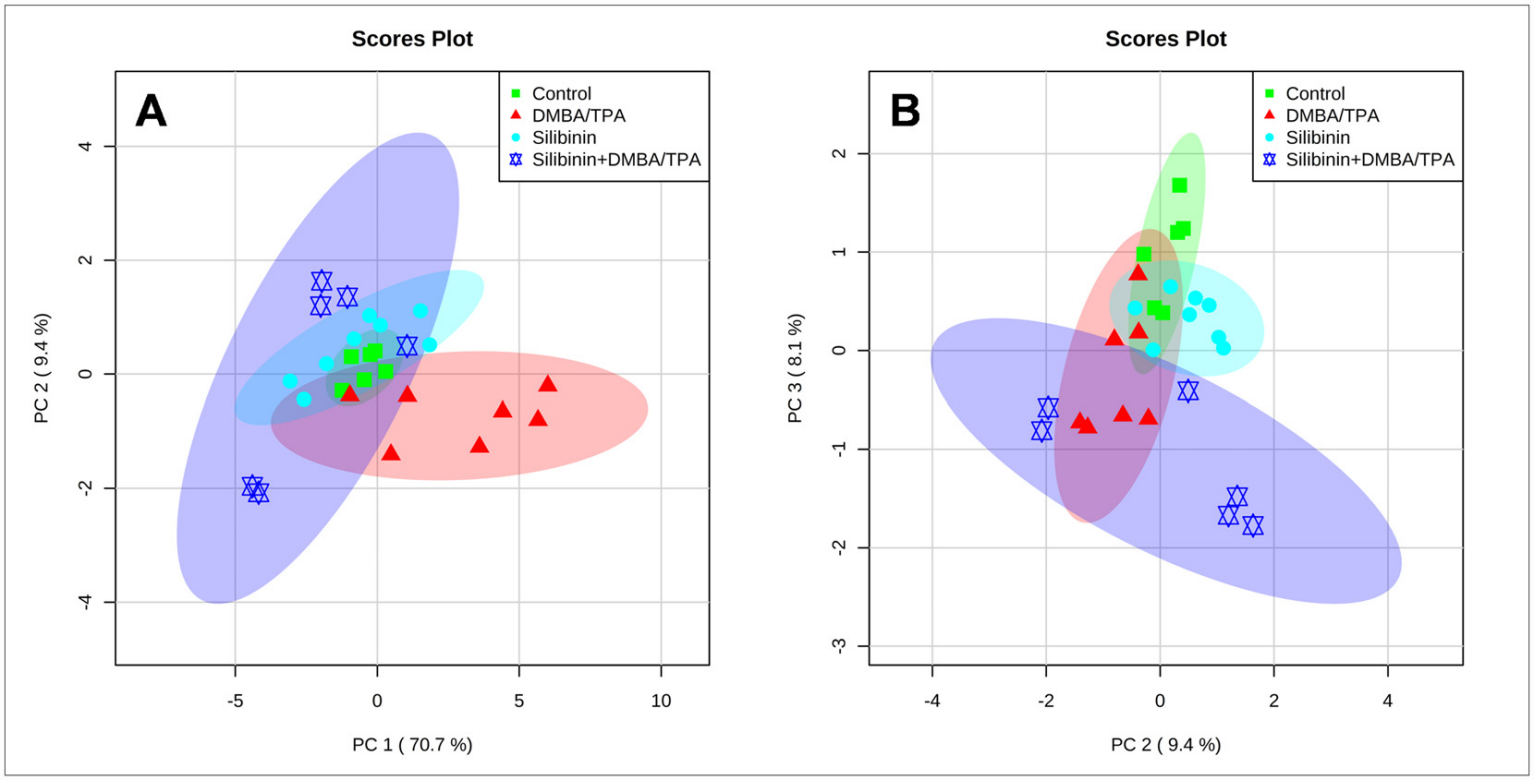
PCA scores plot (A) PC 1 vs PC 2; (B) PC 2 vs PC 3 for the data set consisting of concentrations of 22 metabolites in the hydrophilic extracts of mice skin tissues from all the treatment groups. The percentage of variance explained by each PC is marked. The ellipses enclosing each data set describe 95% confidence regions. PCA score plots were generated after pareto scaling pre-treatment with each symbol representing the metabolic data set from one animal. The green squares represent the untreated animals (control), red triangles represent the animals treated with DMBA and TPA (DMBA/TPA), cyan circles represent the Silibinin treated group, while blue stars represent the Silibinin+DMBA/TPA treated group.

The unsupervised principal component analysis of the metabolite data from all samples revealed the general structure of the complete data set. First three principal components, together, accounted for 88.2% of the total variance (PC 1: 70.7%, PC 2: 9.4% and PC 3: 8.1%). The PCA results were represented as scores plots demonstrating the clustering of samples. (Fig 8) The samples with similar metabolic profile grouped together whereas the samples with altered metabolism were found to be dispersed. In the PC 1 vs PC 2 scores plot (Fig 8A), the control and Silibinin groups reflected similar metabolic profiles with control group being a subset of the Silibinin group. The DMBA/TPA group showed considerable difference from the control group by increased PC 1 scores and decreased PC 2 scores. The Silibinin+DMBA/TPA group differs from the DMBA/TPA group along both the PC 1 and PC 2 axes. For Silibinin+DMBA/TPA group, the PC 1 scores were less than the DMBA/TPA group. Moreover, the Silibinin+DMBA/TPA group showed higher spread along the PC 2 axis. In the PC 2 vs PC 3 scores plot (Fig 8B), the control and DMBA/TPA groups were found to be spanning along the PC 3 component with control group having higher scores. In the Silibinin and Silibinin+DMBA/TPA groups, Silibinin administration shifts the metabolic profile more towards PC 2. Thus, as a result of Silibinin administration, PC 2 appears to be responsible for the separation between the control and the Silibinin groups as well as the DMBA/TPA and the Silibinin+DMBA/TPA groups.

**Fig 9:**
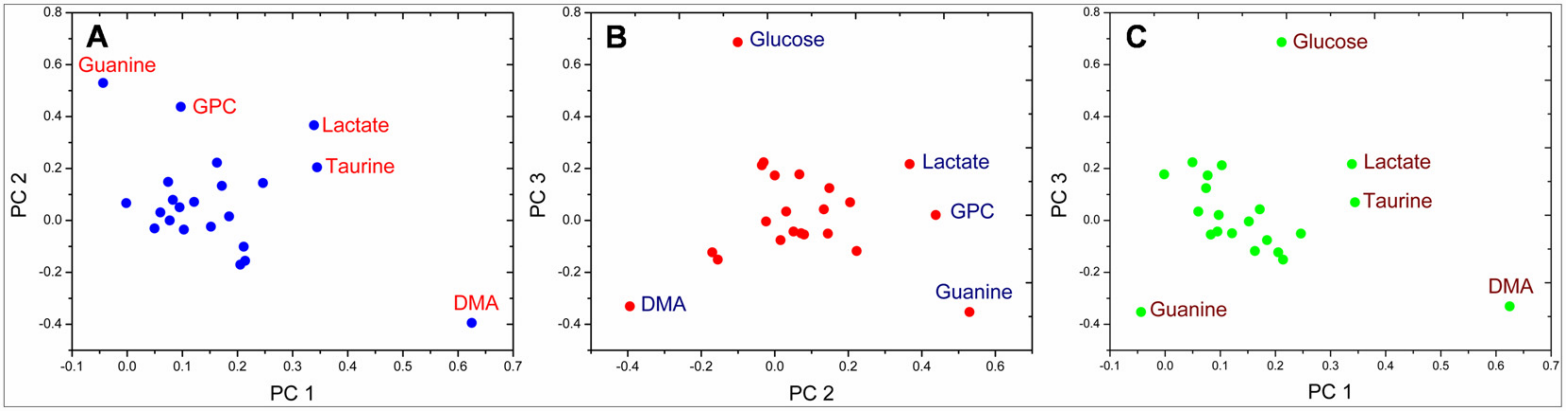
Loadings plots (A) PC 1 and PC 2; B) PC 1 and PC 3; C) PC 2 and PC 3 for the data set consisting of concentrations of 22 metabolites in the hydrophilic skin tissue extracts from mice of all the treatment groups with each data point representing one metabolite. In the loading plots A), B) and C), name of the metabolites those were primary contributors to the separation of groups recognized in the PCA are marked alongside the respective data point.

**Fig 10:**
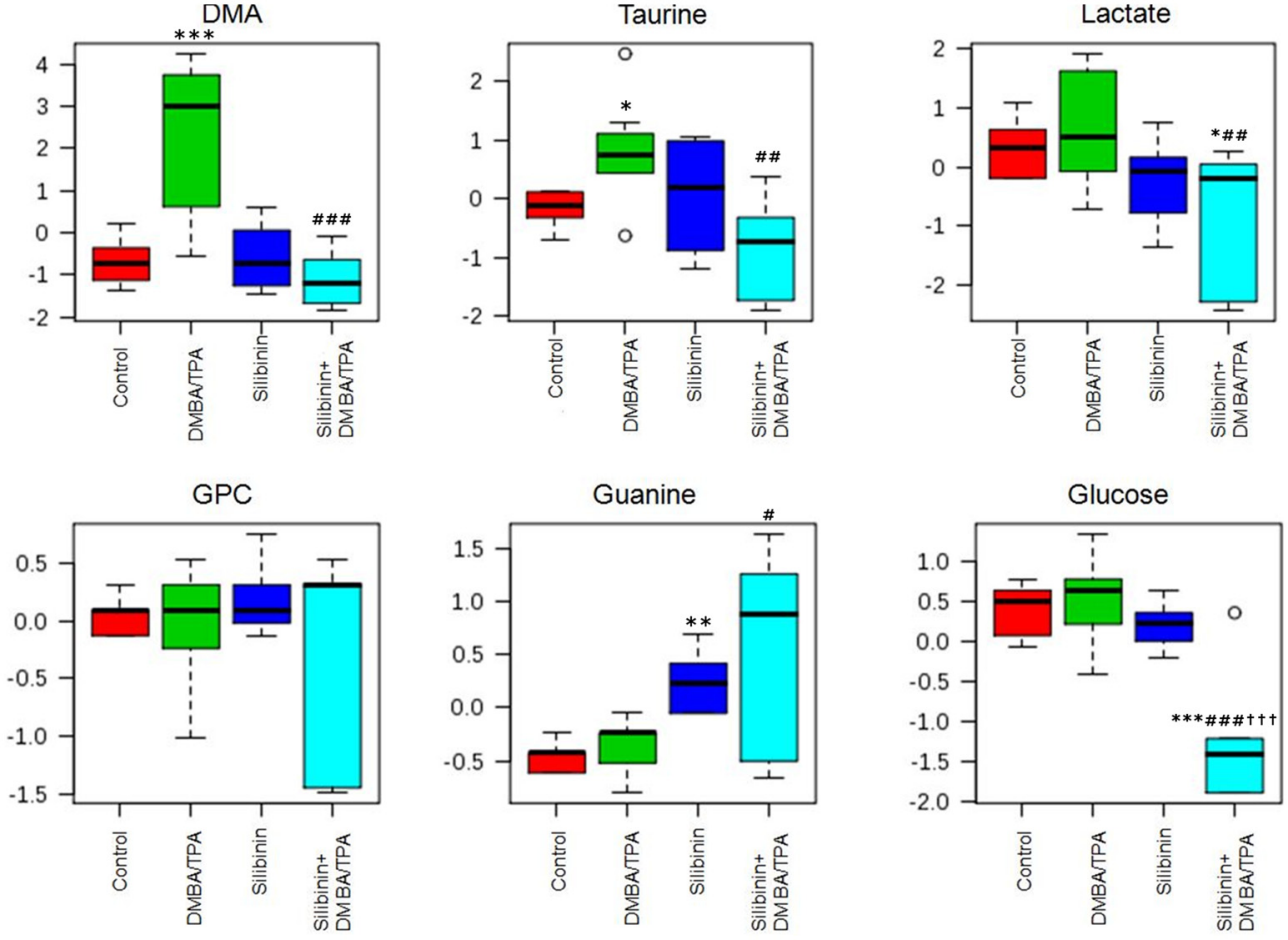
Box and whisker plots displaying the normalized concentration of skin tissue metabolites in control, DMBA/TPA, Silibinin and Silibinin+DMBA/TPA treated groups respectively. Data are expressed as mean±SD and are analyzed by one-way ANOVA followed by post hoc test. * (p<0.05), ** (p<0.01) and *** (p<0.001) indicate significant change with respect to the control group; ^#^ (p<0.05), ^##^ (p<0.01) and ^###^ (p<0.001) indicate significant change with respect to the DMBA/TPA treated group; ^†††^ (p<0.001) indicate significant change with respect to the Silibinin treated group.

The loadings plot between PC 1 and PC 2 displays guanine, Glycerophosphocholine (GPC), lactate, taurine and dimethylamine (DMA) as the major contributors for group separation. The loadings plot between PC 1 and PC 3 shows glucose, lactate, taurine, DMA and guanine as the discriminating variables responsible for group separation. Similarly, glucose, lactate, GPC, guanine and DMA are the variables responsible for discrimination between groups as shown in the loadings plot between PC 2 and PC 3. (Fig 9) The box and whisker plots of these six metabolites are depicted in Fig 10 and are discussed separately as follows:

#### DMA

There was a significant increase (p<0.001) in the levels of DMA observed in the DMBA/TPA group as compared to the control group. However, in the Silibinin+DMBA/TPA group, the DMA levels were lowered (p<0.001) with respect to the DMBA/TPA group. There was no change in the DMA content in the control and the Silibinin groups.

#### GPC

No significant intergroup variation was observed in the GPC content in all the treated groups. However, in the Silibinin+DMBA/TPA group, most of the values were dispersed towards lower range relative to the DMBA/TPA group.

#### Taurine

The taurine content was raised (p<0.05) in the DMBA/TPA group as compared to the control group. In the Silibinin+DMBA/TPA group, the amount of taurine was lower (p<0.01) in comparison to the DMBA/TPA group. There was no significant change in the taurine content in the control and the Silibinin groups.

#### Lactic Acid

There was a slight increase in the concentration of lactic acid in the DMBA/TPA group with respect to the control group, but the change was not significant. While, in the Silibinin+DMBA/TPA group, there was a significant decrease in concentration of lactic acid; (p<0.01) as compared to the DMBA/TPA group, and (p<0.05) in comparison to the control group.

#### Glucose

No significant variation in the glucose levels were found in the DMBA/TPA and Silibinin treated groups with respect to the control group. However, in the Silibinin+DMBA/TPA group, significant reduction (p<0.001) was observed in the glucose concentration in comparison to the control, DMBA/TPA and Silibinin groups.

#### Guanine

In Silibinin group, a significant increase (p<0.01) in the guanine levels was observed with respect to the control group. Also, the increase of guanine levels in the Silibinin+DMBA/TPA group were found to be significant (p<0.05) when compared to the DMBA/TPA group.

## Discussion

The chemopreventive potential of topically administered Silibinin on chemically or UV-induced skin tumors has been reported extensively. [18,44–46] Although, this route of administration shows promising effects, but the limitation is that we cannot predict the location of the initiation of skin tumorigenesis. Hence, oral administration, which is convenient and would ultimately reach the systemic circulation may be the most promising route of administration for prophylaxis and was chosen as the mode of drug administration in our study.

Silibinin manifests its role as an antioxidant when administered orally for 2 weeks. This is suggested due to a significant decrease observed in lipid peroxidation levels. An augmentation in the activity of antioxidant enzymes such as GPx, SOD, and Catalase which may possibly be acting as free radical scavengers, thereby lowering the lipid peroxidation levels. [47] [48]

The effect of Silibinin on the antioxidant defense mechanism and its role on the extent of oxidative stress during the initiation of promotion stage (end of 10 weeks) of carcinogenesis have been investigated in the present study. At the end of this period, histopathological analysis reveals that administration of Silibinin in DMBA/TPA group, results in reduced infiltration of epidermal cells towards dermis as compared to the DMBA/TPA group. This may be due to a moderate increase in LPO levels observed in Silibinin+DMBA/TPA treated group which leads to transient or permanent cell cycle arrest and induces cell differentiation. [49] The increase in LPO levels in the Silibinin+DMBA/TPA group is associated with a decrease in the GSH content which is consistent with previous observations from our laboratory. [10]

During the promotion stage of skin tumorigenesis (end of 22 weeks), LPO levels in the DMBA/TPA group were significantly lowered. The low levels of ROS are reflected to be mitogenic and promotes cell proliferation and survival, [49] which explains the above finding. Whereas, elevated LPO levels in Silibinin treated tumors implicate the pro-oxidant behavior of Silibinin resulting in the decrease in tumor volume in comparison to the DMBA/TPA group. Additionally, the histological analysis of the Silibinin+DMBA/TPA treated tumor showed the presence of empty spaces, which also support the decrease in tumor volume observed. Similar to present findings, the pro-oxidant nature of *A*. *indica* in tumors has also been observed from our laboratory and others. [8,10,50] However, it has also been reported [46] that topical administration of Silibinin hinders cutaneous lipid peroxidation. This antagonist behavior of Silibinin could be due to the difference in the mode of administration of the drug, due to which its absorption into the systemic circulation is affected.

Choline and its derivatives are the main components of phospholipid metabolism of cell membranes and have been identified as markers of cellular proliferation. [51] Choline is transformed to methylamines such as Trimethylamine (TMA), Trimethylamine N-oxide (*TMAO*) and DMA co-processed by gut microbiota and mammalian enzyme systems. [52] Alternatively, phosphorylation of choline by choline kinase produces phosphorylcholine. [53] The DMA level in the DMBA/TPA group is considerably increased in comparison to the control group. Similar to our observations, an increase in the TMAO levels was also reported in oral squamous cell carcinoma. [54] However, in the Silibinin+DMBA/TPA group, the DMA level is significantly lower with no change observed in the GPC content; thereby showing a significant role of Silibinin in restoring the altered choline metabolism observed in the DMBA/TPA group.

Taurine (2-aminoethanesulfonic acid), is an antioxidant and strongly scavenges the hydroxyl radical implying its protective role in shielding various tissues from the oxidative stress generated by xenobiotics exposure. [55] The concentration of taurine has been reported to be elevated in case of squamous cell carcinoma [56] prostate cancer and liver metastasis. [57] The lowering of LPO levels in the DMBA/TPA group in the present study can be associated with an enhanced free radical scavenging due to the increase in taurine levels as observed through ^1^H NMR spectroscopy. Whereas, in the Silibinin+DMBA/TPA group, the taurine concentration is significantly lowered in comparison to the DMBA/TPA group. This is suggestive of increased oxidative stress due to Silibinin treatment which is reflected by the increase in LPO levels. Other reports on various pathologies have divulged a similar physiological role of taurine in lowering oxidative stress. [58–60]

Tumor cells shift to a high rate of aerobic glycolysis instead of depending on oxidative phosphorylation for generation of ATP. This anomalous behavior is commonly referred as the Warburg effect giving rise to the elevated levels of lactic acid and glucose uptake. [61,62] ^1^H NMR metabolomics data revealed no appreciable change in the lactate and glucose levels in the DMBA/TPA group as compared to the control group suggesting no obvious Warburg effect in the tumorous skin. Consistent with our findings, in oral squamous cell carcinoma, no upregulation of the lactate levels were observed suggesting the absence of Warburg effect. [54]

If glucose metabolism remains inadequate, the cells can switch to mitochondrial oxidation of fatty acids and amino acids, which are energy rich but do not readily support cell growth and lead to potentially dangerous levels of reactive oxygen species. [63] In the Silibinin+DMBA/TPA treated group, a significant reduction in the glucose concentration might have resulted into an increased rate of apoptosis and elevated LPO levels. The lowering of glucose levels in the Silibinin+DMBA/TPA group is indicative of a biochemical target for selectively increasing cytotoxicity and oxidative stress in the cancer cells.

We further tried to explore the mode of cell death in the DMBA/TPA induced tumors and its modulation, if any, by Silibinin. The Silibinin treated DMBA/TPA group reveals enhanced apoptosis in tumors in comparison to the DMBA/TPA group. The increased apoptosis may also be responsible for the decrease in the size of the tumors in the Silibinin+DMBA/TPA group. *In vitro* studies on the human keratinocyte HaCaT cells have also reported that Silibinin elevates oxidative stress and thus induces apoptosis. [64] The induction of apoptotis by Silibinin, as a chemopreventive response in UV induced skin tumorigenesis has also been reported earlier. [18,65,66]

Guanine converts into 8-oxoguanine under increased oxidative stress, resulting in buildup of single strand breaks (SSBs) in the nascent DNA strand. The increase of Guanine in the Silibinin+DMBA/TPA group in comparison to the DMBA/TPA group might have led to the accumulation of 8-oxoguanine in the nuclear DNA resulting in poly (ADP-ribose) polymerase (PARP) to bind to the SSBs thus forming poly (ADP-ribose) polymer (PAR). The continuous stimulation of PARP causes exhaustion of both nicotinamide adenine dinucleotide (NAD^+^) and ATP, leading to nuclear translocation of apoptosis inducing factor (AIF), thus causing programmed cell death. This hypothesis also gets support from our TUNEL assay results, where increased apoptosis was a prominent feature observed in Silibinin treated tumors. [67]

In conclusion, the oral administration of Silibinin in the normal and tumor treated skin tissue exhibits a dual nature. It behaves as an antioxidant at the pre-initiation stage followed by its pro-oxidant role in the post-initiation and promotion stages of skin carcinogenesis. The elevated LPO levels displaying pro-oxidant nature of Silibinin in regressing tumors are buttressed by the increase in guanine levels along with decreased taurine and glucose levels observed using ^1^H NMR spectroscopy. Additionally, the enhanced oxidative stress might have led to the programmed cell death in Silibinin treated tumors, thus resulting in significant lowering of tumor volume as well as tumor burden.

## Supporting Information

S1 Fig **(A)** The liquid chromatographic profile of 50 ppm Silibinin solution. The peaks from two diastereoisomers of Silibinin are well resolved with retention time of 3.18 and 4.12 minutes respectively. **(B)** The total ion current (TIC) profile during LC-ESI-MS of 50 ppm Silibinin solution. The peaks at 3.46 and 4.46 minutes correspond to the peaks at 3.18 and 4.12 minutes in the LC chromatogram respectively, due to the time lag between LC and ESI. **(C) and (D)** ESI-MS spectra of 50 ppm Silibinin solution show intense peaks at m/z 481 corresponding to the LC retention time of 3.18 (C) and 4.12 (D) respectively thus validating the elution and separation of Silibinin isomers.
(DOCX)

S2 Fig ESI-MS spectrum of plasma from untreated mouse (control). (DOCX)

S3 Fig ESI-MS spectrum of plasma from untreated mouse spiked with 1 ppm concentration of Silibinin (positive control). (DOCX)

S4 Fig ESI-MS spectrum of plasma after single Silibinin dose (500 mg/kg body weight in 0.5% CMC, per oral) at 5 minutes post treatment. (DOCX)

S5 Fig ESI-MS spectrum of plasma after single Silibinin dose (500 mg/kg body weight in 0.5% CMC, per oral) at 10 minutes post treatment. (DOCX)

S6 Fig ESI-MS spectrum of plasma after single Silibinin dose (500 mg/kg body weight in 0.5% CMC, per oral) at 30 minutes post treatment. (DOCX)
