# Supplementary material for: Pro-Oxidant Role of Silibinin in DMBA/TPA Induced Skin Cancer: 1H NMR Metabolomic and Biochemical Study
Source: PLoS One. 2016 Jul 14;11(7):e0158955. doi: 10.1371/journal.pone.0158955 (PMC4944989; doi:10.1371/journal.pone.0158955)
Supplement: S1 Fig — (A) The liquid chromatographic profile of 50 ppm Silibinin solution. The peaks from two diastereoisomers of Silibinin are well resolved with retention time of 3.18 and 4.12 minutes respectively. (B) The total ion current (TIC) profile during LC-ESI-MS of 50 ppm Silibinin solution. The peaks at 3.46 and 4.46 minutes correspond to the peaks at 3.18 and 4.12 minutes in the LC chromatogram respectively, due to the time lag between LC and ESI. (C) and (D) ESI-MS spectra of 50 ppm Silibinin solution show intense peaks at m/z 481 corresponding to the LC retention time of 3.18 (C) and 4.12 (D) respectively thus validating the elution and separation of Silibinin isomers. (DOCX) [file pone.0158955.s001.docx]

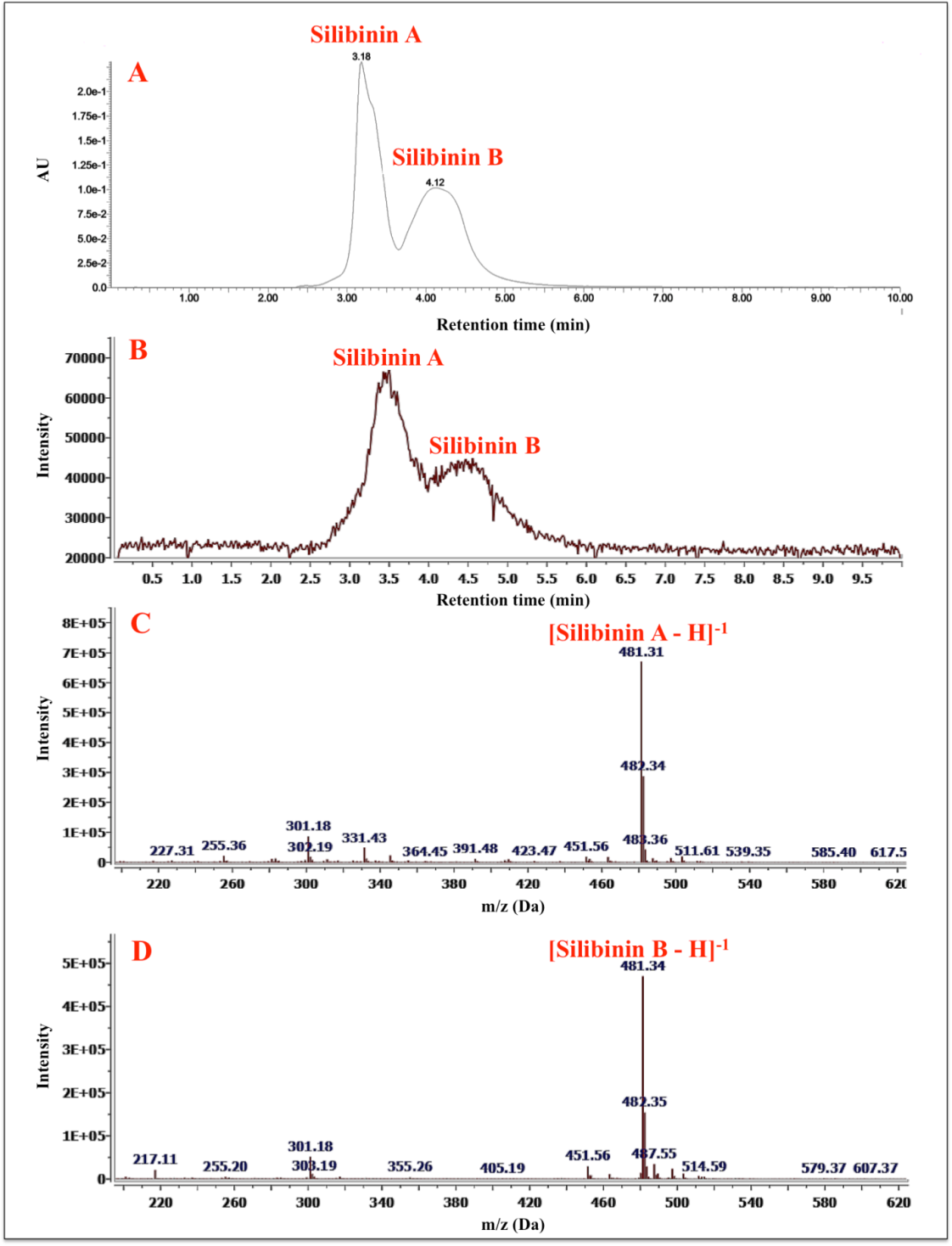


**S1 (A) Fig** The liquid chromatographic profile of 50 ppm Silibinin solution. The peaks from two diastereoisomers of Silibinin are well resolved with retention time of 3.18 and 4.12 minutes respectively. **S1 (B) Fig** The total ion current (TIC) profile during LC-ESI-MS of 50 ppm Silibinin solution. The peaks at 3.46 and 4.46 minutes correspond to the peaks at 3.18 and 4.12 minutes in the LC chromatogram respectively, due to the time lag between LC and ESI. **S1 (C and D) Figs** ESI-MS spectra of 50 ppm Silibinin solution show intense peaks at m/z 481 corresponding to the LC retention time of 3.18 (C) and 4.12 (D) respectively thus validating the elution and separation of Silibinin isomers.
