# Supplementary material for: Pro-Oxidant Role of Silibinin in DMBA/TPA Induced Skin Cancer: 1H NMR Metabolomic and Biochemical Study
Source: PLoS One. 2016 Jul 14;11(7):e0158955. doi: 10.1371/journal.pone.0158955 (PMC4944989; doi:10.1371/journal.pone.0158955)
Supplement: S2 Fig — (DOCX) [file pone.0158955.s002.docx]

**S2 Fig**. ESI-MS spectrum of blood plasma from untreated mouse (control).
