# Supplementary material for: Pro-Oxidant Role of Silibinin in DMBA/TPA Induced Skin Cancer: 1H NMR Metabolomic and Biochemical Study
Source: PLoS One. 2016 Jul 14;11(7):e0158955. doi: 10.1371/journal.pone.0158955 (PMC4944989; doi:10.1371/journal.pone.0158955)
Supplement: S4 Fig — (DOCX) [file pone.0158955.s004.docx]

**S4 Fig.** ESI-MS spectrum of blood plasma after single Silibinin dose (500 mg/kg body weight in 0.5% CMC, per oral) at 5 minutes post treatment
